# Editorial: Molecular mechanism of neuroimmune modulation and synaptic plasticity in acute and chronic pain

**DOI:** 10.3389/fnmol.2023.1224792

**Published:** 2023-06-09

**Authors:** Linlin Zhang, Xin Zhang, Zilong Wang, Yize Li

**Affiliations:** ^1^Department of Anesthesiology, Tianjin Medical University General Hospital, Tianjin, China; ^2^Tianjin Research Institute of Anesthesiology, Tianjin, China; ^3^Center for Translational Pain Medicine, Department of Anesthesiology, Duke University Medical Center, Durham, NC, United States; ^4^Department of Anesthesiology, The Affiliated Wuxi People's Hospital of Nanjing Medical University, Wuxi, Jiangsu, China; ^5^SUSTech Center for Pain Medicine, School of Medicine, Southern University of Science and Technology, Shenzhen, China

**Keywords:** pain, dorsal root ganglion, neuroinflammation, astrocyte, microglia, synaptic plasticity, sleep deprivation

As editors of this Research Topic, it was our pleasure to collect a wide range of fascinating articles and reviews to further our understanding of novel pain-associated molecules and their signaling pathways, which can be used as therapeutic targets for acute and chronic pain treatment. In this editorial we recapitulate the major findings and perspectives detailed within each of the accepted articles.

In the past decades, we have witnessed exciting discoveries that the interaction between neurons and glial cells contributes to peripheral and central sensitization of nociceptive circuitry, which governs multiple nociceptive perception and excitatory synaptic transmission. However, the specific molecular and cellular mechanisms underlying neuroimmune modulation, synaptic plasticity, and pain behaviors remain elusive and attract considerable interests. Cheng et al. summarized the latest findings that astrocyte activation and the alternations in the molecular cascades (such as intracellular kinases, channels, receptors, and transcription factors) and mechanisms involved in the spinal dorsal horn and supraspinal structures are dominant steps for the initiation and persistence of neuropathic pain. Li X. et al. reported that 147 differentially expressed mRNAs (136 upregulated and 11 downregulated) in the medial prefrontal cortex (mPFC) of rats with bone cancer pain using high-throughput sequencing of the transcriptome. Among them, MHCII in the mPFC may be a key biomarker for microglia activation and neuroinflammation to facilitate antigen processing and further evoke bone cancer pain. Toll like receptor 9 (TLR9) is an important sensor for danger-associated molecular patterns (DAMPs) and an indispensable effector of non-sterile/sterile inflammation among all TLRs. Chen Y. et al. found the robust increase of TLR9 mRNA and decrease of paw withdrawal mechanical threshold in CFA-treated mice. Both TLR9 antagonism and neuronal TLR9 downregulation in the spinal cord elevated paw withdrawal mechanical threshold after complete Freund's adjuvant (CFA) injection, demonstrating the involvement of neuronal TLR9 in the spinal cord in CFA-induced inflammatory pain. Great attentions have been recently paid to emphasizing and criticallydiscussing the cGAS-STING cascades, which is required for the elimination of pathogens and damaged host cells during innate and tumor immunity, as well as inflammatory diseases. Wu et al. summarizes the advances made in identifying the mechanisms underlying the involvement of cGAS-STING cascades in primary sensory neurons and glia cells in the regulation of chronic pain with different etiologies, such as low back pain, bone cancer pain, fractures-associated postoperative pain, chemotherapy-induced neuropathy and nerve injuries-induced neuropathic pain, but it continues to be incomprehensible about our understanding of its diverse functions on chronic pain.

The activation of excitatory pain-related proteins and receptors is a cardinal feature of nociceptive synaptic plasticity and sensory neuronal excitability. TMEM100, a two-transmembrane protein, was recently identified as an effector to disinhibition of TRPA1 activity in sensory neurons. Wang P. et al. reported that TMEM100 co-expressed with TRPA1 and TRPV1 in trigeminal ganglion neurons-innervating the temporomandibular joint and masseter muscle and their up-regulation of co-expressions was detected following temporomandibular disorder pain induced by the inflammation of temporomandibular joint or the trauma of masseter muscles. Furthermore, specific deficiency of TMEM100 in trigeminal ganglion neurons or local treatment with TMEM100 inhibitor into the temporomandibular joint or masseter muscles reduced temporomandibular disorder pain and TRPA1 activation in trigeminal ganglion neurons. Collectively, these intriguing findings demonstrate that TMEM100 in trigeminal ganglion neurons causes temporomandibular disorder pain via modulating the activity of TRPA1 within the TRPA1-TRPV1 complex. A study by Li W. et al. observed several dorsal root ganglion (DRG) neurons innervating the ST36 acupoint and ipsilateral hind paw plantar using retrograde tracing, chemogenetic, morphological, and behavioral experiments. Suppressing these shared neurons attenuated CFA-induced inflammatory pain in mice, and elevated the mechanical pain threshold of ST36 acupoint in the CFA model. Moreover, most of the shared DRG neurons express TRPV1, a marker of nociceptive neurons. These results indicate that the shared nociceptive DRG neurons participate in ST36 acupoint sensitization in CFA-induced chronic pain. Huang et al. review the recent pathogenesis of synapses dysfunction by Shank3 regulation in Autism spectrum disorder (ASD)-related nociceptive paresthesia. Also, Li Z. et al. review recent literature focusing on menthol-related drugs for pathologic pain management in clinical trials, especially in neuropathic pain, cancer pain, musculoskeletal pain, as well as postoperative pain, with the purpose to search the novel therapeutic candidates for pain resolution in clinics.

To achieve molecular and neurobiological insights into the peripheral sensory neurons in chemotherapy-induced neuropathic pain, Mao et al. utilized transcriptomic analysis to profile mRNA and non-coding RNA expression in the DRGs of mice receiving paclitaxel treatment. They detected 372 differentially expressed genes in the DRGs of paclitaxel-treated mice. Among them, there were 8 mRNAs, 3 long non-coding RNAs (lncRNAs), 16 circular RNAs (circRNAs), and 345 microRNAs (miRNAs). More interestingly, they compared the expression levels of differentially expressed miRNAs and mRNA in the DRGs of mice with paclitaxel-induced neuropathic pain against those evaluated in other models of neuropathic pain induced by other chemotherapeutic agents, nerve trauma, or diabetes. There are dozens of shared differentially expressed miRNAs between paclitaxel and diabetes, but only a few shared miRNAs between paclitaxel and nerve trauma. Simultaneously, there is no shared differentially expressed mRNA between paclitaxel and nerve trauma. These identify that differentially expressed genes in DRGs vary greatly among neuropathic pain with different etiologies. Guo et al. discovered that *Rmst* (rhabdomyosarcoma 2-associated transcript) as a lncRNA was specifically expressed *Atf3*^+^ injured DRG neurons and considerably increased following peripheral nerve trauma. Disrupting the expression of *Rmst* in injured DRGs ameliorated nerve trauma-caused allodynia and blocked *Dnmt3a* (DNA methyltransferase 3 alpha) expression, revealing the pivotal roles of *Rmst* for neuropathic pain development.

Patients with pain often experience insomnia, depression, anxiety and cognitive impairments, which is known to be associated with worsening pain and a serious threat to their quality of life. One study by Chen S. et al. reported that one night of sleep deprivation is sufficient to increase nociceptive sensitivity and cause oxidative insult in rats and humans. One night of recovery sleep restored basal nociceptive sensitivity in rats and improved the sleep deprivation-induced increase in pain intensity in volunteers, but with a slight protection against oxidative damage. Yet, it remains largely unclear whether sleep deprivation-induced hyperalgesia is associated with oxidative stress. Another study by Abdul et al. showed that ventral tegmental area glutamatergic neurons with projections to nucleus accumbens are important in chronic constrictive injury (CCI)-induced neuropathic pain and CFA-induced inflammatory pain and pain-induced anxiety and depression, providing a promising mechanism for developing novel therapeutic methods. Also, Shen et al. gave an overview on the structural functions implicated in the nociceptive circuitry and advanced emotional cortex circuitry. They further emphasize the current insights into sex dimorphism in neuromodulation of pain and related mental disorders via endogenous dopamine, 5-hydroxytryptamine, GABAergic inhibition, norepinephrine, and peptide pathways like oxytocin, as well as their receptors, which is critical for improving individualized medical care.

## Author contributions

All authors listed have made a substantial, direct, and intellectual contribution to the work and approved it for publication.

